# Novel SLC16A2 mutations impair thyroid hormone transport and drive neurodevelopmental deficits in Chinese patients with allan-herndon-dudley syndrome

**DOI:** 10.1038/s41598-026-40703-3

**Published:** 2026-03-01

**Authors:** Xiaoang Sun, Chao Wang, Longlong Lin, Xiaoping Lan, Shengnan Wu, Xuqin Chen, Cheng Cai

**Affiliations:** 1 Department of Neurology, Shanghai Children’s Hospital, School of Medicine, Shanghai Jiao Tong University, Shanghai, China; 2 Department of Clinical Laboratory, Shanghai Chlidren’s Hospital, School of Medicine, Shanghai JiaoTong University, Shanghai, China; 3 Department of Neonatology, Shanghai Children’s Hospital, School of Medicine, Shanghai Jiao Tong University, Shanghai, China

**Keywords:** Allan-Herndon-Dudley syndrome, SLC16A2, MCT8, thyroid hormone, mental retardation, Diseases, Endocrinology, Genetics, Neuroscience

## Abstract

This study elucidates the molecular pathogenesis of neurodevelopmental deficits in Chinese Allan-Herndon-Dudley syndrome (AHDS) patients caused by pathogenic SLC16A2 mutations. Genetic analysis of three Han Chinese patients with severe intellectual disability and global developmental delay identified two novel truncating mutations (c.1093del [p.A365Lfs35] and c.270_271del [p.G91Lfs28]) and a hemizygous de novo splice-site mutation (c.1026 + 1G > A). Structural modeling predicted that the c.1093del variant causes C-terminal truncation of transmembrane helix 12, which is likely to disrupt the T3-binding pocket by impairing the critical Arg445–His415 hydrogen bond. Functional studies confirmed significantly reduced SLC16A2 expression (*P* < 0.05) accompanied by dysregulated thyroid metabolism (increased *DIO2* and *HR*; *P* < 0.01) and downregulated neurodevelopmental genes (*Nrgn* and *KIF9*; *P* < 0.001). Mechanistically, MCT8 deficiency impaired cerebral thyroid hormone uptake, driving synaptic and axonal defects through dysregulation of both transcriptional and cytoskeletal programs: T3-dependent transcriptional suppression via inactivation of the *Nrgn* promoter thyroid response element, and disruption of the *KIF9* signaling axis. These findings establish novel genotype-phenotype correlations in Chinese AHDS patients and provide a mechanistic framework for understanding the neurodevelopmental consequences of impaired thyroid hormone transport, with patient-derived iPSCs serving as a valuable resource for future therapeutic development.

**Supplementary Information:**

The online version contains supplementary material available at 10.1038/s41598-026-40703-3.

## Introduction

Allan-Herndon-Dudley syndrome (AHDS) is an X-linked disorder characterized by severe intellectual disability and a distinctive thyroid hormone profile, caused by mutations in the solute carrier family 16 member 2 (*SLC16A2)* gene^1,2^. *SLC16A2* (Homo sapiens NM_006517.3, NP_006508.2) encodes monocarboxylate transporter 8 (MCT8, MIM#300095), situated on the X chromosome (Xq13.2) and comprising 6 exons and 5 introns, spanning a total length of 112.6 kb^3^. Since its initial description in 1944, over 150 mutations in the *SLC16A2* gene have been identified in patients with AHDS, including missense, nonsense, deletion, insertion, frameshift, and splice site mutations^4,5^. The majority of affected individuals are male, with an estimated incidence rate of approximately 1 in 70,000^6^. Clinical hallmarks encompass a spectrum of neurological impairments: severe intellectual disability; poor head control; difficulties or inability to sit, stand, or walk independently; axial hypotonia; absence of speech; progression from hypotonia to spastic paraplegia; chorea; epilepsy; and delayed myelination. Additionally, patients with AHDS exhibit abnormal serum thyroid function characterized by elevated triiodothyronine (T3) levels, low-normal thyroxine (T4) levels, decreased reverse T3 (rT3) levels, and normal to elevated TSH levels. Most heterozygous female carriers of *SLC16A2* gene mutations typically show no clinical symptoms but may present minor abnormalities on thyroid function tests^7^.

The severe neurological sequelae of AHDS primarily attributed to cerebral hypothyroidism caused by defective neuronal T3 uptake via MCT8^8–10^. Consequently, the expression of key thyroid hormone (TH)-responsive effector genes in the brain is expected to be profoundly altered.While this transport defect is established, the precise molecular pathways downstream of MCT8 deficiency that lead to specific neurodevelopmental deficits remain incompletely understood. To bridge this gap, investigating key transcriptional targets of TH signaling, which serve as both functional biomarkers and mechanistic mediators, is crucial. For instance, Type 2 iodothyronine deiodinase (*Dio2*) activates T4 to T3 and is feedback-regulated by TH levels, providing a sensitive readout of local TH status. Similarly, the TH-inducible Krüppel-like factor 9 (*KIF9*) and the synaptic plasticity protein Neurogranin (*Nrgn*) are direct T3 target genes essential for neuronal differentiation and circuit maturation. Additionally, the co-repressor Hairless (*HR*) modulates T3 receptor activity, fine-tuning the transcriptional response. Therefore, we hypothesize that the expression patterns of *Dio2*, *KlF9*, *Nrgn*, and *HR* will be significantly dysregulated in a model of MCT8 deficiency. Analyzing these genes provides a direct functional readout of disrupted cerebral T3 signaling and its downstream consequences, forming the mechanistic rationale for this study.

Epidemiological data on AHDS, both globally and within China, remain scarce, and its true incidence is undefined. In this study, we report three sporadic cases of AHDS in Chinese patients, expanding the clinical and mutational spectrum of the disorder. Using next-generation sequencing, we identified a novel splice-site mutation and two previously unreported nonsense mutations in the *SLC16A2* gene, and we proceeded to investigate their functional consequences on thyroid hormone signaling and neurodevelopmental gene expression.

## Materials and methods

### Compliance with ethical standards

This study was reviewed and approved by the Ethics Committee of Shanghai Children’s Hospital (approval No.2019R071-F03). Informed consent was obtained from the parents of the patients for the use of their blood samples in genetic analysis. Written informed consent to participate in this study and for publication purposes was provided by the legal representatives of the participants. The study adhered to Chinese bioethics laws as well as the Helsinki Declaration and its subsequent amendments.

### Exome sequencing

Exome sequencing was performed on the proband and her parents, as previously described^11^. Briefly, genomic DNA was isolated from peripheral blood and sheared using a Covaris Ultra Sonicator. Exonic regions were captured with the IDTxGen Exome Research Panel (IDT), and sequencing was carried out on an Illumina HiSeq 2500 platform to generate 150-bp paired-end reads. Reads were aligned to the human reference genome (GRCh37/hg19) using the Burrows-Wheeler aligner (BWA, v0.7.15)^12^. Variant calling for single-nucleotide variants (SNVs) and indels was performed using Samtools^13^ and Pindel^14^. Following annotation with Ingenuity Variant Analysis, common variants (with a minor allele frequency ≥ 0.05) were filtered out.

### Real-time quantitative PCR (qPCR) analysis of *SLC16A2* expression and its interacting complexes

Total RNA was extracted from peripheral blood mononuclear cells (PBMCs) of participants using the QIAGEN RNA Preparation Kit. cDNA was synthesized using the PrimeScript™ RT Master Mix (Takeda). Quantitative PCR (qPCR) was performed on a LightCycler^®^ 96 system (Roche) with SYBR Premix Ex Taq II (Takeda), in accordance with the manufacturers’ instructions. The primer sequences used are listed in Supplementary Table S1. RT-qPCR was utilized to evaluate the relative expression levels of *SLC16A2*, *DIO2*, *HR*, *Nrgn*, and *KIF9* within the nuclear family of patients as well as in age- and sex-matched control groups. Each dataset for transcription levels was generated from three independent experiments, with results presented as mean ± standard error. Statistical analysis of qPCR data was conducted using the 2-ΔΔCt method to compare average values among samples. Asterisks indicate statistically significant differences (one-way ANOVA; **P < 0.05, ***P < 0.01).

### In silico pathogenicity and evolutionary conservation analysis

To assess the pathogenicity of the identified variants, we employed a tiered approach using specialized algorithms based on variant type. For the prediction of the impact of loss-of-function variants (nonsense and frameshift mutations) and the splice-site mutation, we primarily relied on MutationTaster^15^. which is designed to handle these variant classes. As part of its analysis, MutationTaster incorporates evolutionary conservation metrics. A positive PhyloP score indicates evolutionary conservation (with higher values denoting stronger conservation), while a negative score suggests a non-conserved region. Similarly, PhastCons scores (ranging from 0 to 1) indicate higher conservation as the value approaches 1.To further and independently evaluate sequence conservation at the mutation sites, we performed a parallel analysis using the UCSC Genome Browser (Human Dec. 2013, GRCh38/hg38 assembly). This allowed us to visually confirm and contextualize whether the mutated positions on both alleles were located within evolutionarily conserved genomic regions.

### Protein structure prediction of nonsense mutations using the I-TASSER server

The I-TASSER Suite pipeline comprises four key steps: threading template identification, iterative structure assembly simulation, model selection and refinement, and structure-based function annotation. The server can be accessed at http://zhanglab.ccmb.med.umich.edu/I-TASSER.

### Distribution of variants in *SLC16A2*

We mapped the locations of newly identified variants to their corresponding domains and analyzed the distribution of other reported variants.

### Patient-specific iPSC generation and characterization

Peripheral blood mononuclear cells (PBMCs) from three AHDS patients carrying pathogenic *SLC16A2* variants were reprogrammed using non-integrating Sendai virus vectors (CytoTune-iPS 3.0; expressing OCT4, SOX2, KLF4, c-MYC)^16^. Established iPSC clones were expanded in mTeSR Plus medium on Matrigel-coated plates. Comprehensive pluripotency validation included: Morphology: Bright-field imaging confirmed typical pluripotent stem cell colony architecture (high nuclear-cytoplasmic ratio, defined borders).Flow cytometry: Quantification of pluripotency markers (OCT4, SOX2, NANOG, TRA-1-60, SSEA4; >95% positive population). Trilineage differentiation: In vitro spontaneous differentiation via embryoid body formation, with immunostaining for ectoderm (βIII-TUBULIN), mesoderm (a-SMA), and endoderm (AFP) lineages.Karyotyping: G-banding analysis confirmed genomic integrity (20 metaphase spreads per line).STR profiling: STR MultiAmplification Kit (PowerPlex 21D System, Promega, Madison, WI, USA) verified genetic match to parental PBMCs. Analyzer (Applied Biosystems, Foster City, CA, USA). STR analysis was performed by Guangzhou Cellcook Biotech Co., Ltd. (Guangzhou, China).

## Results

### Clinical case

Three male patients presenting with global developmental delay were evaluated in the pediatric neurology department. Their clinical characteristics are summarized in Table 1. Patient 1 was admitted at 12 months, Patient 2 presented at 10 months, and Patient 3 was referred at 9 months. All had unremarkable perinatal histories, and their parents were non-consanguineous. Patient 1 had a positive family history: a maternal half-brother died of cerebral palsy. All patients exhibited severe motor delay (poor head control, inability to sit independently), absent speech, and reduced responsiveness.

**Fig. 1:**
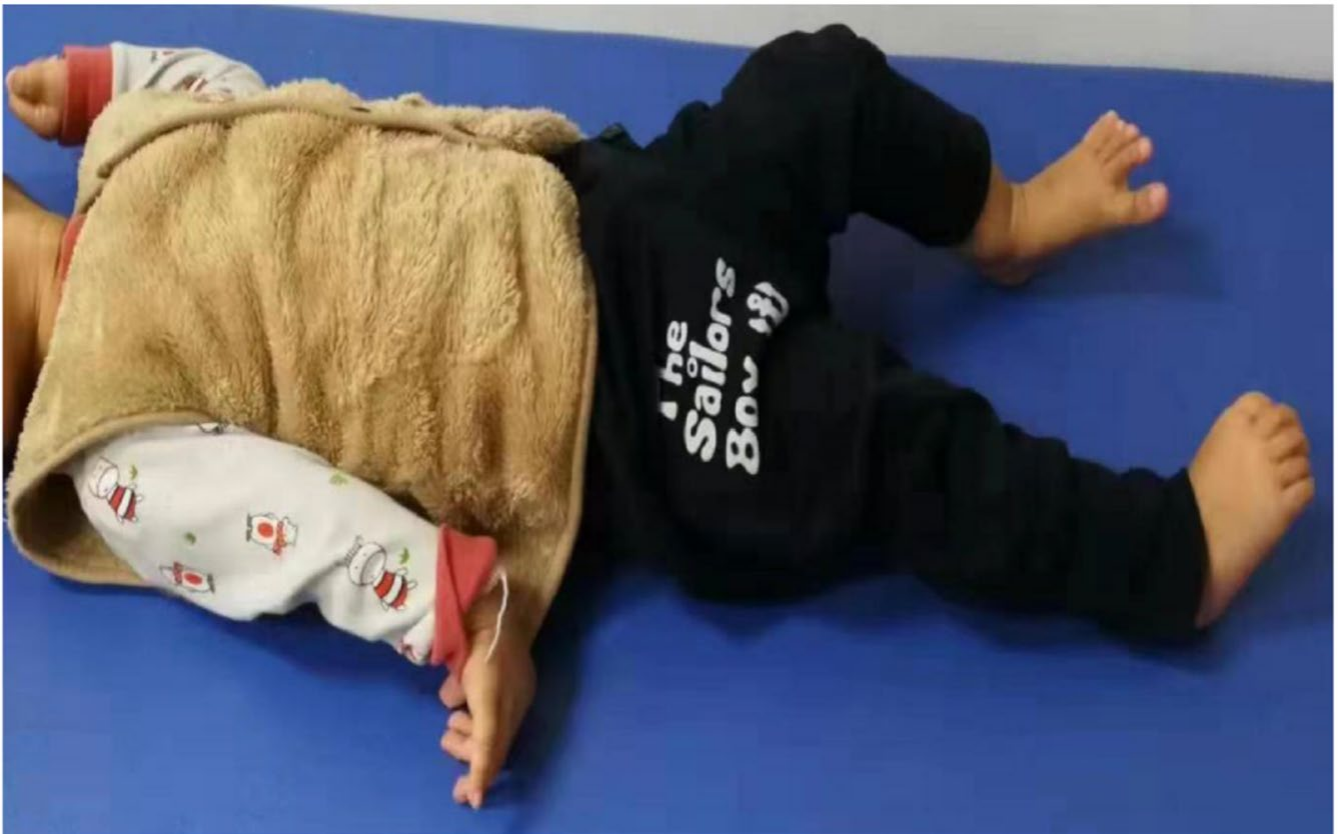
Clinical photograph of Patient 1, depicting abnormal posture.

Notably, Patient 1 displayed spastic paralysis and atypical craniofacial features (Fig. 1). Serum thyroid function tests revealed the characteristic AHDS profile: elevated fT3 and low fT4 with normal TSH in Patient 1 (Table 1). Routine laboratory tests were normal. Electroencephalography (EEG) was abnormal only in Patient 2. Brain MRI showed delayed myelination in Patients 2 and 3, and bilateral paraventricular white matter changes in Patient 1.

**Table 1:**
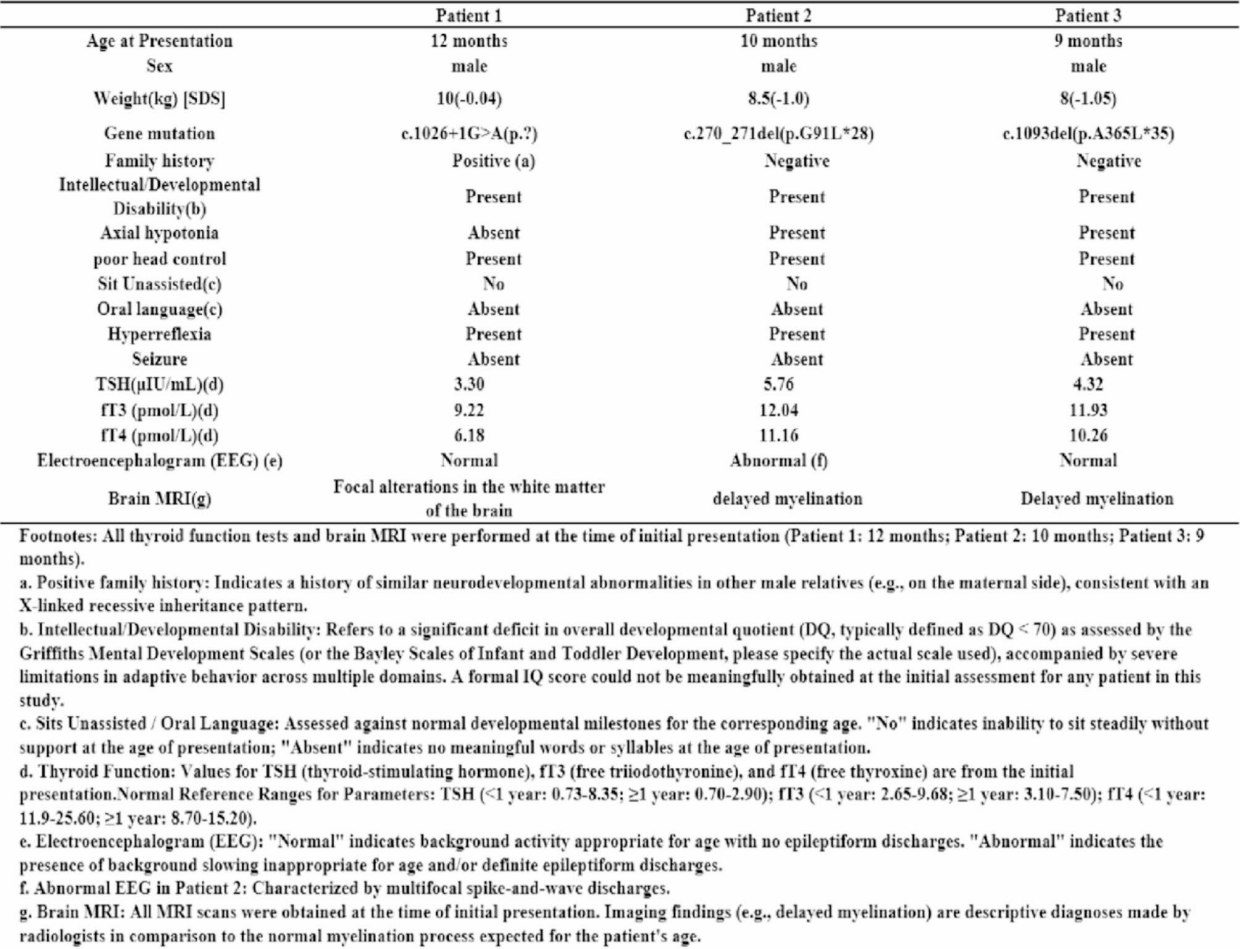
Clinical manifestations and mutation types in 3 patients with AHDS caused by SLC16A2 mutations.

### Gene sequencing analysis

**Fig. 2:**
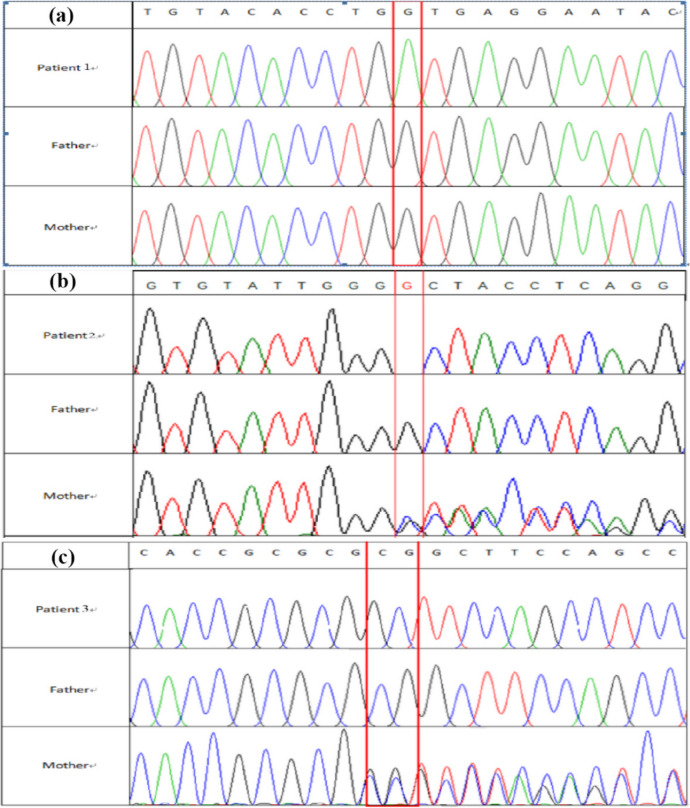
(**a**)The sequences of genomic DNA in the patient’s nuclear family with the detection of a hemizygous de novo splicing variant of *SLC16A2* c.1026 + 1G > A in patient (1) (**b**)The sequences of genomic DNA in the patient’s nuclear family with the detection of a novel hemizygous nonsense variant of *SLC16A2* c.270_271del(p.G91L*28) in patient (2) (**c**)The sequences of genomic DNA in the patient’s nuclear family with the detection of a novel hemizygous nonsense variant of *SLC16A2* c.1093del(p.A365L*35) in patient 3.

Genetic analysis identified pathogenic SLC16A2 mutations in all patients (Fig. 2a-c). Patient 1 harbored a hemizygous de novo splice-site mutation (c.1026 + 1G > A), absent in both parents and unreported in population databases (ACMG classification: pathogenic). Patient 2 carried a hemizygous nonsense mutation (c.270_271del; p.G91Lfs28) inherited from his mother. Patient 3 carried a maternally inherited hemizygous nonsense mutation (c.1093del; p.A365Lfs35).

### RT­qPCR analysis of *SLC16A2* expression and its interacting complexes

**Fig. 3:**
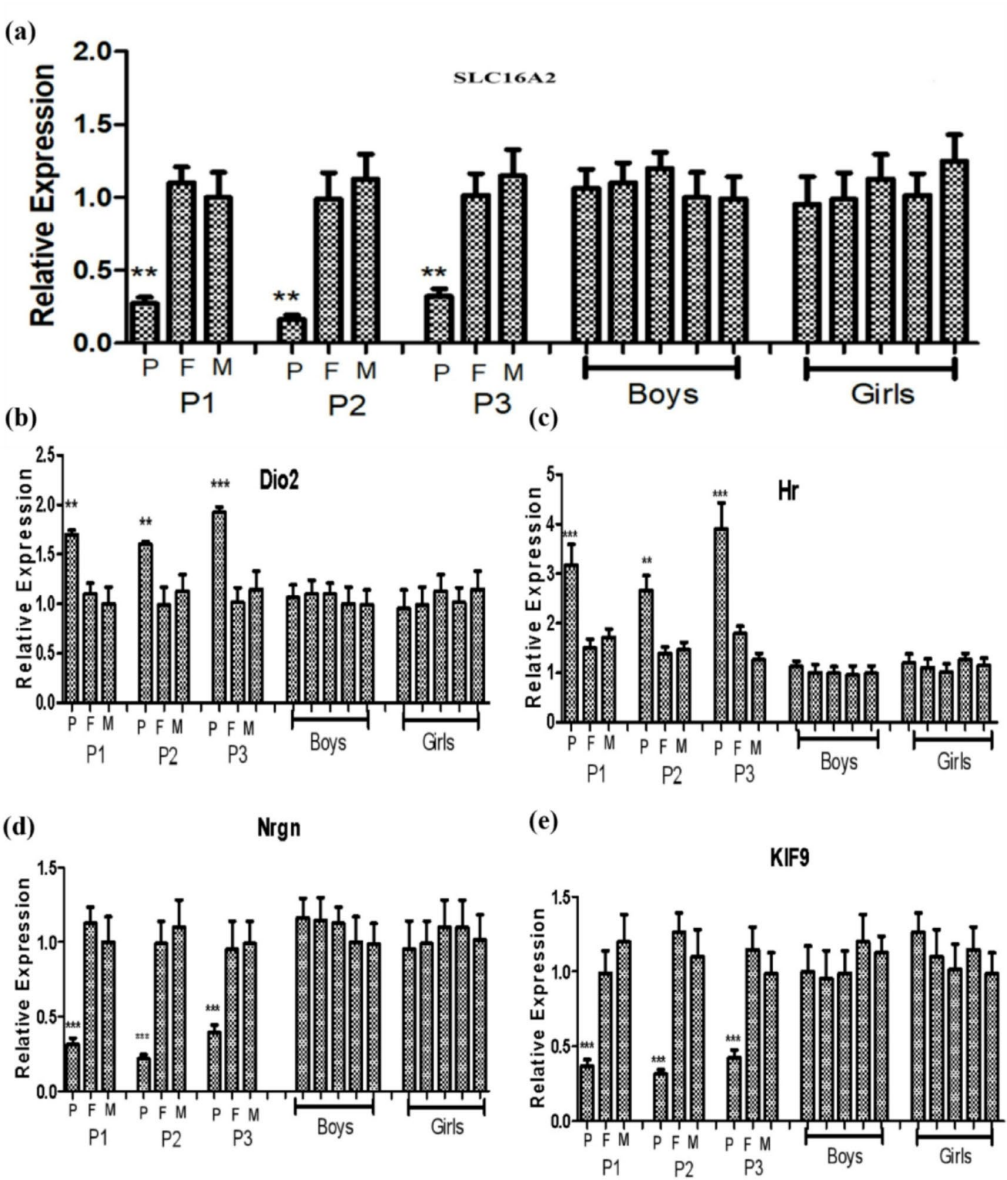
A comparative analysis of the transcriptional products of the *SLC16A2* gene and its interacting genes across three familial lineages and ten normal controls. (**a**)Real-­time quantitative polymerase chain reaction experimentswere performed for *SLC16A2* mRNA expression in the patient’s nuclear family. *SLC16A2* mRNA expression levels in patients were significantly different from those of their parents and normal children of different sexes. (**b**–**f**) The mRNA expression of the interacting complexes were analyzed. The *DIO2*, *HR*, *Nrgn* and *KIF9* expression levels in the children were significantly different from those of their parents and healthy children of different sexes. ***p < 0.01.

We next assessed the expression of *SLC16A2* expression and its interacting complexes and compared them to their parents and age/sex-matched healthy controls using RT-qPCR. SLC16A2 expression was significantly lower in all patients compared to controls and parents (*P* < 0.05; Fig. 3a). In contrast, expression of *DIO2* and *HR* was significantly elevated in patients (*P* < 0.05 and *P* < 0.001, respectively; Fig. 3b, c). Expression of the neurodevelopmental genes *Nrgn* and *KIF9* was significantly downregulated in patients (both *P* < 0.001; Fig. 3e, f).

### In silico Pathogenicity and Evolutionary Conservation Analysis

**Fig. 4:**
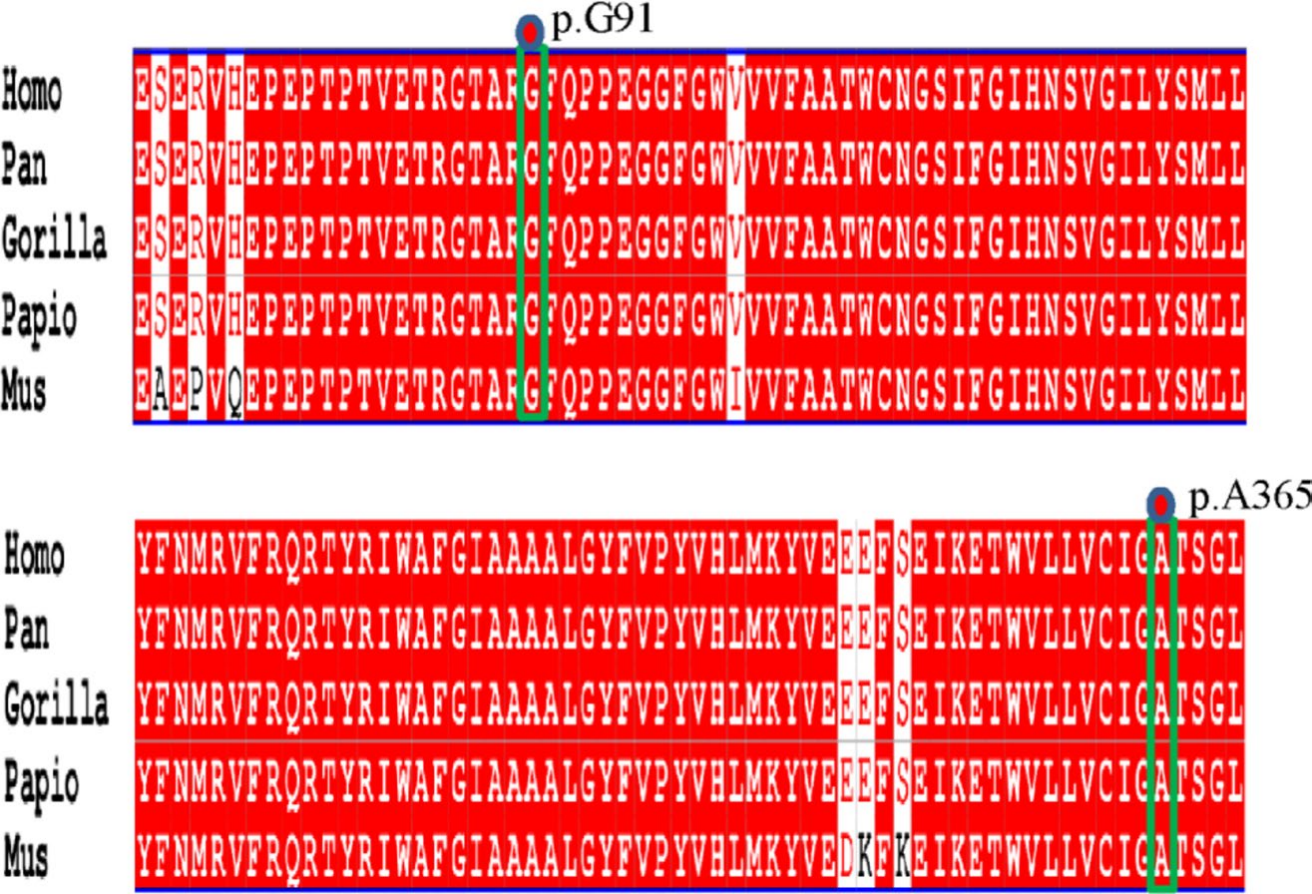
Comparative sequence alignment of the 91st and 365th amino acid residues of the *SLC16A2* gene across various species.

In silico analysis identified three novel variants in the SLC16A2 gene: two frameshift mutations (c.1093del, p.Ala365Leufs35; c.270_271del, p.Gly91Leufs28) and one canonical splice-site mutation (c.1026 + 1G > A). Mutation Taster predicted all three variants to be disease-causing. Evolutionary conservation analysis using PhyloP and PhastCons scores revealed that the nucleotide positions affected by these mutations are located in highly conserved genomic regions (PhyloP > 0, PhastCons ≈ 1). Visual inspection in the UCSC Genome Browser (GRCh38/hg38 assembly) further confirmed the high evolutionary conservation of the sequences surrounding both frameshift mutation sites (Fig. 4).

### Protein structure prediction on the iterative threading assembly refinement (I-TASSER) server for the nonsense mutation

**Fig. 5:**
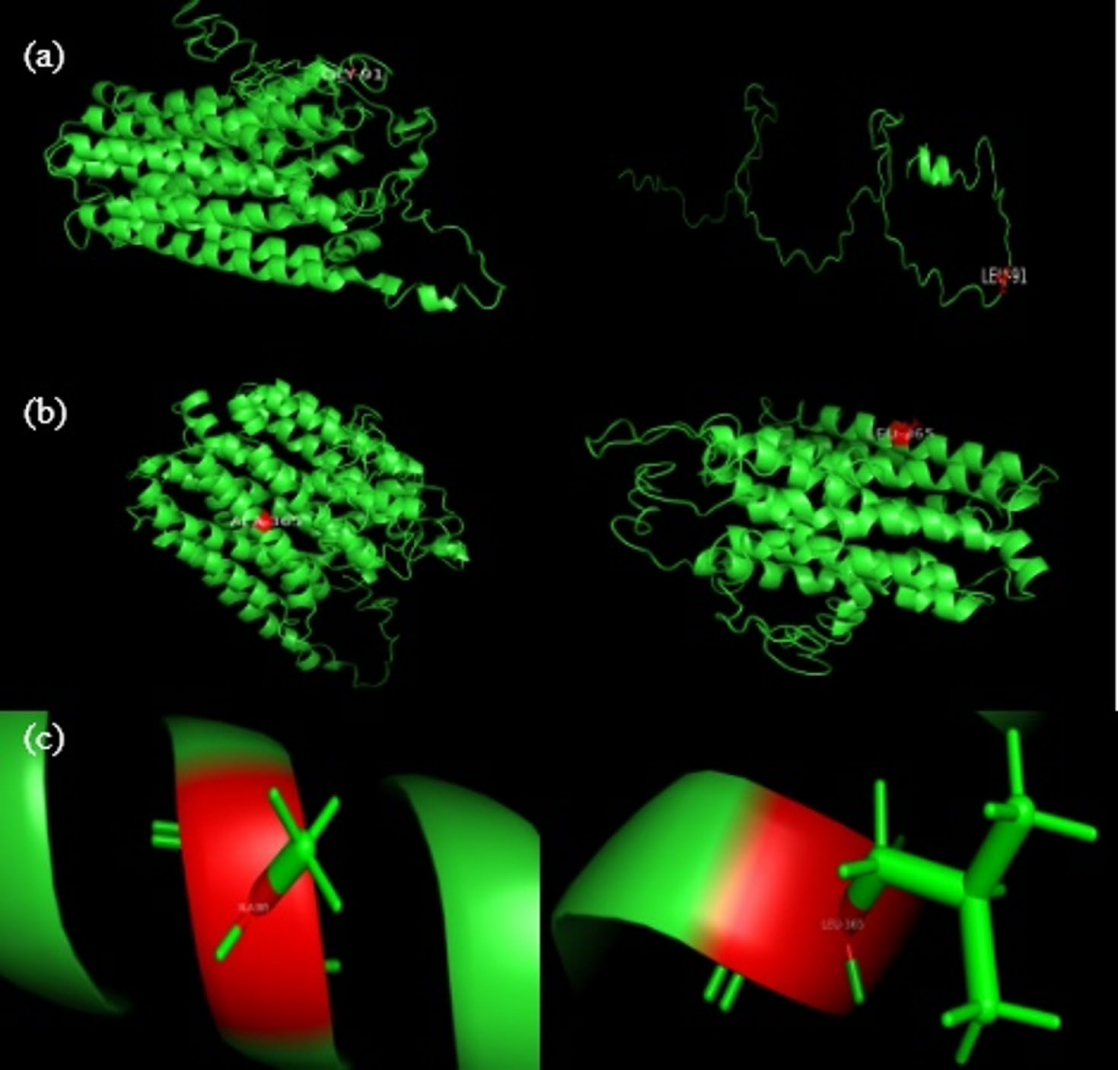
(**a**): Three-dimensional structural model of the wild-type MCT8 protein sequence and the mutant c.270_271del (p.G91L*28); (**b**): The three-dimensional structure models of the wild-type MCT8 protein and the mutant c.1093del(p.A365L*35); (**c**):The helical structure at the 365th amino acid position in the wild-type MCT8 protein and the mutant c.1093del(p.A365L*35).

The iterative threading assembly refinement (I-TASSER) server generated three-dimensional (3D) atomic models based on protein sequences from the novel nonsense mutation of c.1093del(p.A365L*35) in *SLC16A2*, which showed that residue 365 was located in the ligand binding sites(Fig. 5).

### Distribution of variants in *SLC16A2*

**Fig. 6:**
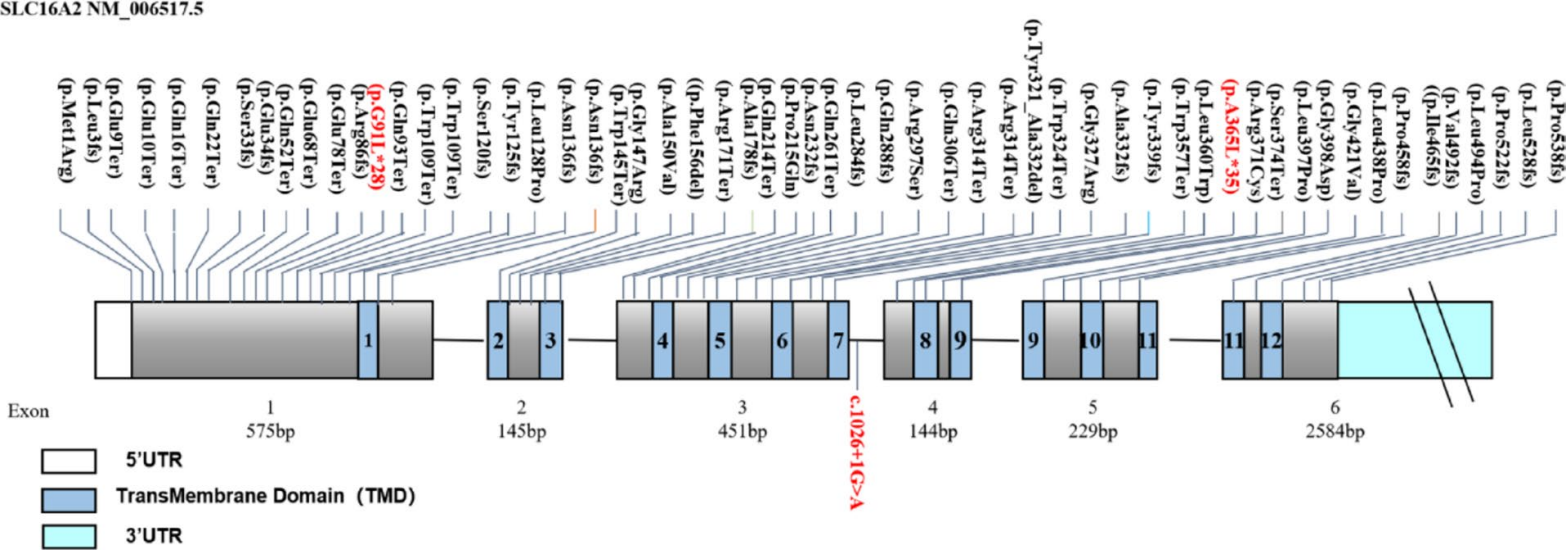
Distribution of pathogenic variants in *SLC16A2*. The novel pathogenic variants in our study are shown in red; previously reported variants are displayed in black.

This gene encodes a protein composed of 539 amino acids, featuring 12 transmembrane domains. The majority of the gene is situated within exons 1 and 3, as illustrated in Fig. 6.

### Generation and characterization of the patient-specific iPSC line SHCDNi003-A

The SHCDNi003-A iPSC line was derived from the patient’s PBMCs using non-integrating Sendai virus vectors carrying OCT3/4, SOX2, KLF4, and c-MYC. The line exhibited pluripotency markers (OCT4, TRA-1-60, SSEA4) and trilineage differentiation potential (PAX6/Brachyury/AFP). Karyotype analysis confirmed a normal 46, XY genotype. The patient-specific *SLC16A2* mutation (c.1026 + 1G > A) was retained, with STR identity verification. Mycoplasma testing was negative^16^.

### Discussion

This study identified novel loss-of-function SLC16A2 mutations in three Chinese AHDS patients, which were associated with the classic severe neurological phenotype of the disorder. Cranial MRI findings in all patients, including paraventricular white matter changes and delayed myelination, were consistent with the established AHDS phenotype as reported in the literature for loss-of-function *SLC16A2* variants^17,18^. These characteristic neuroimaging features support the severe neurological phenotype observed in our cohort. Notably, Patient 1 displayed the characteristic serum thyroid profile of elevated fT3 and low fT4 with normal TSH. These variants are predicted to cause complete loss of MCT8 function via nonsense-mediated decay (NMD) or protein truncation^19^. Structurally, MCT8, a member of the major facilitator superfamily (MFS), transports thyroid hormones via an alternating access mechanism, whereby its two 6-transmembrane helix bundles undergo rigid-body rotation to alternate between outward- and inward-open conformations^20^. The truncating mutations identified herein would disrupt this essential architecture, abolishing thyroid hormone transport. Collectively, our findings expand the mutational and clinical spectrum of AHDS in the Han Chinese population and establish a clear genotype-phenotype correlation for protein-truncating variants.

Mutations in SLC16A2, encoding MCT8, were established as causative for AHDS in 2004^1^. MCT8 is a crucial thyroid hormone transporter, especially vital in the brain^21–23^. Our patients exhibited 100% severe phenotypes, aligning with reports that the majority of AHDS cases present with severe-to-profound disability^4^. This contrasts with milder phenotypes occasionally associated with hypomorphic missense variants in some cohorts^24,25^. Our findings are consistent with the known gradient of phenotypic severity dictated by the SLC16A2 genotypic spectrum, where truncating mutations, as identified here, confer the most severe neurological impairment^26^.

The profound neurological severity in our cohort is consistent with the predicted complete loss of MCT8 transporter function caused by the identified nonsense and splice-site mutations. This causal link between transporter ablation and severe neurodevelopmental deficits is robustly supported by preclinical models, such as the Mct8/Oatp1c1 double-knockout mouse, which recapitulates key features including cerebral hypomyelination and motor impairment due to central hypothyroidism^27^.Consequently, MCT8 dysfunction leads to cerebral hypothyroidism, which is central to AHDS pathology^21–23^.

The pathogenicity of the identified *SLC16A2* variants is supported by converging lines of in silico and genetic evidence, which bridge the gap between genotype and the severe clinical phenotypes observed in our patients. First, the two frameshift variants (c.1093del, p.Ala365Leufs35; c.270_271del, p.Gly91Leufs28) and the canonical splice-site variant (c.1026 + 1G > A) were all absent from population databases and were private to the affected families. As illustrated in Fig. 6, these variants are located within critical protein-coding exons of the *SLC16A2* gene, which encodes a protein with 12 transmembrane domains. Their occurrence in these structurally vital regions supports their potential to disrupt function. Second, the c.1026 + 1G > A variant’s location at a canonical intron-exon boundary (Fig. 6) strongly predicts disruption of normal splicing, which would likely alter the transmembrane domain architecture. Third, evolutionary conservation analysis revealed that the nucleotide positions affected by these mutations reside in highly conserved genomic regions. As shown in Fig. 4, the Ala365 and Gly91 residues affected by the frameshift mutations are conserved across distant species, underscoring their functional indispensability. This extreme conservation implies that the p.Ala365Leufs35 and p.Gly91Leufs28 frameshifts are highly deleterious to protein function. Collectively, these genetic distribution and bioinformatic analyses—spanning population frequency, protein domain mapping, splice prediction, and evolutionary constraint—lead to a coherent mechanistic hypothesis: these variants disrupt intracellular TH transport, critically reducing T3 availability in neurons and impairing thyroid hormone receptor (TR)-mediated signaling^28,29^.

Our investigation into downstream effects revealed disrupted expression of key T3-responsive genes. Genes such as *Nrgn* (also known as RC3) harbor a functional thyroid hormone response elements (TREs) located in the first intron, confirming it as a direct target of thyroid hormone in the human brain^30^. The diminished neuronal T3 levels resulting from MCT8 dysfunction compromise effective ligand binding to TRs, which impairs their capacity to appropriately modulate the expression of downstream target genes, including *KIF9* and *Nrgn* itself. The observed dysregulation of *DIO2*, *HR*, *Nrgn*, and *KIF9* thus provides a functional readout of impaired cerebral T3 signaling^31–33^. Specifically, the upregulation of *DIO2* likely represents a compensatory attempt to enhance local T3 production from the limited intracellular T4 pool^34^, whereas the altered expression of *HR*, *Nrgn*, and *KIF9* reflects the disruption of transcriptional programs critical for synaptic plasticity and myelination. In particular, the dysregulation of *Nrgn* — a schizophrenia risk gene encoding neurogranin — has been directly shown to impair hippocampal long-term potentiation and disrupt the phosphorylation landscape of postsynaptic proteins, providing a mechanistic link to synaptic plasticity deficits^35^.

This adaptive increase in *DIO2* expression, however, must be interpreted within the context of the irreversible systemic thyroid profile characteristic of AHDS. The observed transcriptional upregulation likely represents a compensatory neuronal response aimed at maximizing local T3 production from the limited intracellular T4 pool, yet it is demonstrably insufficient to normalize thyroid hormone signaling or reverse the systemic biochemical signature. Crucially, this local compensation does not override the characteristic decrease in serum rT3, which is a direct consequence of altered peripheral thyroid hormone metabolism stemming from the MCT8 transport defect.While T4 uptake into the brain may be partially preserved through compensatory transporters such as OATP1C1, circulating T4 concentrations are often subnormal, reducing its overall availability^36^.

This altered signaling milieu is expected to impair astrocyte function, given the established role of T₃ in promoting astrocyte differentiation and maturation^37^, is expected to impair astrocyte function, thereby disrupting their synaptic support functions for neurons. Consistent with this, TH deficiency directly disrupts the glutamatergic system, altering the expression of astrocytic glutamate transporters (GLAST and GLT-1) and impairing synaptic homeostasis^38^. Furthermore, thyroid hormone promotes OPC migration, differentiation, and remyelination, a process involving upregulation of *KIF9* and highly vulnerable to disruption^39,40^. Ultimately, these multifaceted disruptions across neuronal, astrocytic, and oligodendroglial compartments compromise neural development and function.

While our study provides compelling genetic evidence, it is important to note that its primary focus was on establishing the genetic etiology, patient-derived peripheral blood, and downstream transcriptional consequences of the novel *SLC16A2* mutations in a clinical cohort context. While our qPCR data robustly demonstrate a significant reduction in *SLC16A2* mRNA levels, which is a common consequence of nonsense-mediated decay for truncating mutations and splicing defects, we acknowledge that further mechanistic validation at the protein and direct transport-function level would strengthen the functional assignment. Definitive confirmation of absent or truncated MCT8 protein via Western blot, assessment of its subcellular localization via immunofluorescence, and direct measurement of impaired T3 uptake in vitro represent crucial next steps. These experiments, however, typically require the generation of stable isogenic cell lines or patient-derived primary neurons, which were beyond the primary scope of this initial clinical-genetic and pathophysiological correlation study. Our findings of concomitant dysregulation in the established T3-responsive genes *DIO2*, *HR*, *Nrgn*, and *KIF9* in patient-derived cells provide strong indirect functional evidence of impaired T3 signaling resulting from these mutations. Future work utilizing the patient-derived iPSCs generated in this study will be ideally suited to perform these detailed protein biochemical and functional transport assays, thereby building directly upon the genotype-phenotype correlations established here.

Conclusions.

In summary, we report three novel *SLC16A2* mutations associated with severe AHDS in Chinese patients. The identified nonsense and splice-site variants lead to complete MCT8 loss-of-function, resulting in cerebral hypothyroidism, disrupted T3-responsive gene expression *(DIO2*,* HR*,* Nrgn*,* KIF9*), and the characteristic severe neurodevelopmental phenotype. These findings broaden the genetic landscape of AHDS and reinforce the correlation between truncating mutations and disease severity. Future research using patient-derived iPSC models will be crucial to further dissect the precise functional consequences and explore potential therapeutic avenues targeting the thyroid hormone signaling pathway in AHDS.

## Supplementary Information

Below is the link to the electronic supplementary material.

Supplementary Material 1

## Data Availability

The datasets generated and/or analysed during the current study are available in the manuscript, the ClinVAR repository, and the accession number is https://www.ncbi.nlm.nih.gov/clinvar/submitters/510185.
