# Supplementary material for: Novel SLC16A2 mutations impair thyroid hormone transport and drive neurodevelopmental deficits in Chinese patients with allan-herndon-dudley syndrome
Source: Sci Rep. 2026 Mar 1;16:11476. doi: 10.1038/s41598-026-40703-3 (PMC13056955; doi:10.1038/s41598-026-40703-3)
Supplement: Supplementary file 1 — Supplementary Material 1 [file 41598_2026_40703_MOESM1_ESM.doc]

Supplementary Table S1. Primer sequences used for quantitative PCR (qPCR).

| Gene Name | Forward Primer | Reverse Primer |
| --- | --- | --- |
| SLC16A2 | CAACATGCGAGTGTTCCGCCAA | AAGAGCACCCAGGTCTCCTTGA |
| Dio2 | TTGAGCCGCTCCAAGTCCACTC | CTGTACTGGAGACATGCACCAC |
| Nrgn | GCGGAAGAAGATAAAGAGCGGAG | GGCATCCATCTCTCCTCGGGA |
| KIF9 | CACGCTCTGAAGGACTCGTTAG | TTGATGGCAGGCTCAGTGGTGA |
| Hr | TCTGCGAACTGCTGGCTTCTAC | GTGCGATAATGCTGTCCAGGATG |

All sequences are shown in the 5' to 3' direction.
